# Outcomes of the EMDataResource Cryo-EM Ligand Modeling Challenge

**DOI:** 10.21203/rs.3.rs-3864137/v1

**Published:** 2024-01-25

**Authors:** Catherine L. Lawson, Andriy Kryshtafovych, Grigore D. Pintilie, Stephen K. Burley, Jiří Černý, Vincent B. Chen, Paul Emsley, Alberto Gobbi, Andrzej Joachimiak, Sigrid Noreng, Michael Prisant, Randy J. Read, Jane S. Richardson, Alexis L. Rohou, Bohdan Schneider, Benjamin D. Sellers, Chenghua Shao, Elizabeth Sourial, Chris I. Williams, Christopher J. Williams, Ying Yang, Venkat Abbaraju, Pavel V. Afonine, Matthew L. Baker, Paul S. Bond, Tom L. Blundell, Tom Burnley, Arthur Campbell, Renzhi Cao, Jianlin Cheng, Grzegorz Chojnowski, Kevin D. Cowtan, Frank DiMaio, Reza Esmaeeli, Nabin Giri, Helmut Grubmüller, Soon Wen Hoh, Jie Hou, Corey F. Hryc, Carola Hunte, Maxim Igaev, Agnel P. Joseph, Wei-Chun Kao, Daisuke Kihara, Dilip Kumar, Lijun Lang, Sean Lin, Sai R. Maddhuri Venkata Subramaniya, Sumit Mittal, Arup Mondal, Nigel W. Moriarty, Andrew Muenks, Garib N. Murshudov, Robert A. Nicholls, Mateusz Olek, Colin M. Palmer, Alberto Perez, Emmi Pohjolainen, Karunakar R. Pothula, Christopher N. Rowley, Daipayan Sarkar, Luisa U. Schäfer, Christopher J. Schlicksup, Gunnar F. Schröder, Mrinal Shekhar, Dong Si, Abhishek Singharoy, Oleg V. Sobolev, Genki Terashi, Andrea C. Vaiana, Sundeep C. Vedithi, Jacob Verburgt, Xiao Wang, Rangana Warshamanage, Martyn D. Winn, Simone Weyand, Keitaro Yamashita, Minglei Zhao, Michael F. Schmid, Helen M. Berman, Wah Chiu

**Affiliations:** [1] Institute for Quantitative Biomedicine, Rutgers, The State University of New Jersey, Piscataway, NJ, USA,; [2] Genome Center, University of California, Davis, CA, USA,; [3] Departments of Bioengineering and of Microbiology and Immunology, Stanford University, Stanford, CA, USA,; [4] Department of Chemistry and Chemical Biology, Rutgers, The State University of New Jersey, Piscataway, NJ, USA,; [5] Rutgers Cancer Institute of New Jersey, Rutgers, The State University of New Jersey, New Brunswick, NJ USA,; [6] San Diego Supercomputer Center, University of California San Diego, La Jolla, CA USA,; [7] Institute of Biotechnology, Czech Academy of Sciences, Vestec, CZ,; [8] Department of Biochemistry, Duke University, Durham NC, USA,; [9] MRC Laboratory of Molecular Biology, Cambridge, UK,; [10] Discovery Chemistry, Genentech Inc, South San Francisco, USA,; [11] Structural Biology Center, X-ray Science Division, Argonne National Laboratory, Argonne, IL, USA,; [12] Structural Biology, Genentech Inc, South San Francisco, USA,; [13] Department of Haematology, Cambridge Institute for Medical Research, University of Cambridge, Cambridge, UK,; [14] Chemical Computing Group, Montreal, Quebec, CA,; [15] Molecular Biophysics and Integrated Bioimaging Division, Lawrence Berkeley National Laboratory, Berkeley, CA, USA,; [16] Department of Biochemistry and Molecular Biology, The University of Texas Health Science Center at Houston, Houston, TX, USA,; [17] York Structural Biology Laboratory, Department of Chemistry, University of York, York, UK,; [18] Department of Biochemistry, University of Cambridge, Cambridge, UK,; [19] Scientific Computing Department, UKRI Science and Technology Facilities Council, Research Complex at Harwell, Didcot, UK,; [20] Center for Development of Therapeutics, Broad Institute of MIT and Harvard, Cambridge, MA, USA,; [21] Department of Computer Science, Pacific Lutheran University, Tacoma, WA, USA,; [22] Department of Electrical Engineering and Computer Science, University of Missouri, Columbia, MO, USA,; [23] European Molecular Biology Laboratory, Hamburg Unit, Hamburg, Germany,; [24] Department of Biochemistry and Institute for Protein Design, University of Washington, Seattle, WA, USA,; [25] Department of Chemistry and Quantum Theory Project, University of Florida, Gainesville, FL, USA,; [26] Theoretical and Computational Biophysics Department, Max Planck Institute for Multidisciplinary Sciences, Göttingen, Germany,; [27] Department of Computer Science, Saint Louis University, St. Louis, MO, USA,; [28] Institute of Biochemistry and Molecular Biology, ZBMZ, Faculty of Medicine and CIBSS - Centre for Integrative Biological Signalling Studies, University of Freiburg, 79104 Freiburg, Germany,; [29] Department of Biological Sciences, Purdue University, West Lafayette, IN, USA,; [30] Department of Computer Science, Purdue University, West Lafayette, IN, USA,; [31] Verna and Marrs McLean Department of Biochemistry and Molecular Biology, Baylor College of Medicine, Houston, TX, USA,; [32] Division of Computing & Software Systems, University of Washington, Bothell, WA, USA,; [33] Biodesign Institute, Arizona State University, Tempe, AZ, USA,; [34] School of Advanced Sciences and Languages, VIT Bhopal University, Bhopal, India,; [35] Electron Bio-Imaging Centre, Diamond Light Source, Harwell Science and Innovation Campus, Didcot, UK,; [36] Institute of Biological Information Processing (IBI-7: Structural Biochemistry) and Jülich Centre for Structural Biology (JuStruct), Forschungszentrum Jülich, Jülich, Germany,; [37] Department of Chemistry, Carleton University, Ottawa, ON, Canada,; [38] Physics Department, Heinrich Heine University Düsseldorf, Düsseldorf, Germany,; [39] Nature’s Toolbox (NTx), Rio Rancho, NM, USA,; [40] Department of Biochemistry and Molecular Biology, University of Chicago, Chicago, IL, USA,; [41] Division of Cryo-EM and Bioimaging, SSRL, SLAC National Accelerator Laboratory, Menlo Park, CA, USA,; [42] Department of Quantitative and Computational Biology, University of Southern California, Los Angeles, CA 90089, USA

## Abstract

The EMDataResource Ligand Model Challenge aimed to assess the reliability and reproducibility of modeling ligands bound to protein and protein/nucleic-acid complexes in cryogenic electron microscopy (cryo-EM) maps determined at near-atomic (1.9–2.5 Å) resolution. Three published maps were selected as targets: *E. coli* beta-galactosidase with inhibitor, SARS-CoV-2 RNA-dependent RNA polymerase with covalently bound nucleotide analog, and SARS-CoV-2 ion channel ORF3a with bound lipid. Sixty-one models were submitted from 17 independent research groups, each with supporting workflow details. We found that (1) the quality of submitted ligand models and surrounding atoms varied, as judged by visual inspection and quantification of local map quality, model-to-map fit, geometry, energetics, and contact scores, and (2) a composite rather than a single score was needed to assess macromolecule+ligand model quality. These observations lead us to recommend best practices for assessing cryo-EM structures of liganded macromolecules reported at near-atomic resolution.

Cryogenic electron microscopy (Cryo-EM) has rapidly emerged as a powerful method for determining structures of macromolecular complexes. It is complementary to macromolecular crystallography in its ability to visualize macromolecules, and complexes thereof, of varying sizes and extents of structural heterogeneity in 3D at near to full atomic resolution. The number of new structures determined by cryo-EM has been steadily increasing, and with improved resolution (Figure 1a). Macromolecular complexes may contain, in addition to larger components (*i.e*., proteins or nucleic acids), smaller components such as enzyme cofactors, substrates, analogs or inhibitors, medically relevant drug discovery candidates or approved drugs, glycans, lipids, ions, or water molecules. Accurate modeling of ligands within their macromolecular environment is important, as they can substantially influence larger-scale structure and functions. As the number of novel ligands in cryo-EM-derived structures continues to increase rapidly (Figure 1b), it becomes important to investigate how best to validate them to ensure optimal modeled ligand quality using various measures such as fit of model-to-map, geometry scores of the ligand, and local interactions with ions, waters, protein or nucleic acid components.

An international workshop on validation of ligands in crystallographic PDB depositions^1^ held in 2015 identified several common problems, including weak experimental density, ligand atoms poorly placed, incorrectly defined or misinterpreted chemical species, and inclusion of atoms not directly supported by experimental evidence. The main outcome was a set of best practice recommendations for PDB depositors and for the PDB archive. For PDB depositors, recommendations included providing unambiguous chemical definitions for all ligands present in a structure, including hydrogen atoms, providing ligand geometry and refinement restraints, clearly identifying atoms not supported by experimental evidence, providing the experimental map used for modeling, and including comments explaining outliers. Recommendations for PDB validation included providing informative images of ligands in their density; providing stick figure diagrams indicating geometry outliers; identifying atoms not supported by experimental evidence; providing quality assessment metrics for each identified ligand; and identifying possible protonation states. Most of the workshop validation recommendations have been implemented in PDB validation reports, with ligand geometric assessments implemented for all experimental methods^2–4^.

Since 2010, EMDataResource (EMDR) has organized multiple Challenge activities (https://challenges.emdataresource.org) with the aim of bringing the cryo-EM community together to address important questions regarding the reconstruction and interpretation of maps and map-derived atomic coordinate models^5^. For each Challenge, a committee consisting of prominent experts is invited to recommend targets and set goals. Each event has been conducted with the operational principles of fairness, transparency, and openness, using modeler-blind assessments and open results, with a major goal of promoting innovation.

In 2016, paired Map and Model Challenges invited participants to apply their novel algorithms/software to reconstruct maps and to evaluate models at resolutions of 2.9–4.5 Å. The results were published in a 19-article special journal issue^6^. By 2018, most participating groups had improved their pipelines, eliminating many identified mistakes. The unique EMRinger map metric for sidechain-mainchain consistency^7^ was first tested systematically in the 2016 Challenge and is now standard.

The 2019 Model Metrics Challenge evaluated models, while also evaluating the effectiveness of many different coordinate-only and map-model fit metrics for 4 targets at 1.7–3.3 Å resolution. The results were published in a single joint paper^8^. To streamline the challenge process, input of data from participants and initial assessment pipelines were automated, and comprehensive statistics, visualizations of scores and comparisons were made available. The CaBLAM multi-residue mainchain metric^9^, introduced in 2016, was shown in the 2019 Challenge to be the score most highly correlated with measures of match-to-target. The Q score^10^, inspired and introduced by the 2019 Challenge, has now been adopted by the wwPDB Validation System used at deposition as well as in the detailed validation report^11^.

The 2021 Ligand Model challenge brought together research and industry groups to evaluate and discuss available measures and tools for ligand quality assessment. Many of the issues identified for crystallographic structures in the 2015 ligand workshop were also expected to occur in cryo-EM structures with modeled ligands, but with additional considerations unique to cryo-EM. Targets were chosen from publicly available maps with sufficient resolution to theoretically allow de-novo ligand modeling, include diverse components such as protein and RNA, and have current interest and relevance. The objectives set out were to identify 1) methods for modeling such ligands and 2) metrics to evaluate map-model fit, stereochemical geometry, and chemically sensible interactions between the ligand and protein or RNA component. We describe here the overall design and outcomes of the EMDR Ligand Challenge, recommendations for the cryo-EM community based on currently available assessment methods, and what is needed for the future.

## Results

### Challenge Design

**Figure 2.**
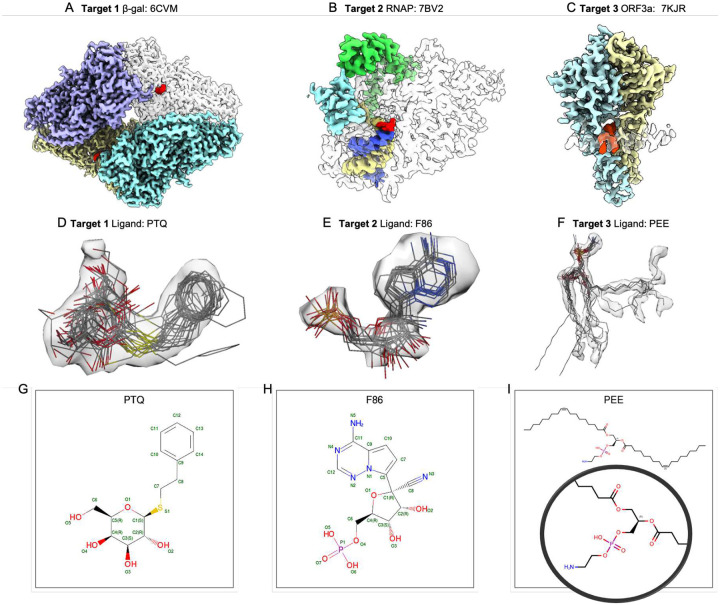
Ligand Challenge targets and ligands from submitted models. In (**A-C**), Targets 1–3 are shown, with each polymer/nucleic acid chain rendered as a separate surface with a different color, in some cases semi-transparent. Target ligands are shown in red. In (**D-F**), segmented density representing each target ligand is shown with a semi-transparent surface, with submitted ligand models overlaid. Map contour levels are 0.35 (2.3σ), 0.036 (2.6σ), 0.25 (3.7σ) respectively (sigma values were calculated from the full unmasked map to capture variation in background noise). (**G-I**) Chemical sketches for each of the target ligands (source: PDB). Selected individual ligand poses from submitted models superimposed on target map densities are shown in Extended Data Figure 1.

Three Cryo-EM map targets were chosen based on the following criteria: recently published with resolution better than 3 Å, maps released in the Electron Microscopy Databank (EMDB), associated coordinates in the Protein Data Bank (PDB), small molecules present (ligands, water, metal ions, detergent, and/or lipid), and having current topical relevance (Figure 2 **panels A-C**):

- **Target 1**: 1.9 Å *E. coli* β-Galactosidase (**β-Gal**) in complex with inhibitor 2-phenylethyl 1-thio-beta-D-galactopyranoside (PETG) with PDB Chemical Composition Dictionary (CCD) id **PTQ**, EMDB map entry EMD-7770, PDB reference model 6CVM^12^
- **Target 2**: 2.5 Å SARS-CoV-2 RNA-dependent RNA polymerase (**RNAP**) with the pharmacologically active, nucleotide form of the prodrug remdesivir (CCD id **F86**) covalently-bound to RNA, EMD-30210, PDB reference model 7BV2^13 14^
- **Target 3**: 2.1 Å SARS-CoV-2 Open Reading Frame 3a (**ORF3a)** putative ion channel in complex with 1,2-dioleoyl-sn-glycero-3-phosphoethanolamine phospholipid (CCD id **PEE**), EMD-22898, PDB reference model 7KJR^15^

Next, modeling teams were solicited via emails to multiple bulletin board lists and were asked to register, generate and upload optimized models for each Target, following provided guidelines (see Online Methods). A total of 61 independently determined models were contributed by seventeen teams from different institutions (ids **EM001-EM017**), with workflow details collected for each (see summary in Table 1 and **Supplementary Data S1, S2** for details).

### Model Assessments

Submitted and PDB reference models for each target were evaluated by passing them through the EMDR Model Challenge validation pipeline^8,16^. Individual scores were obtained for many different sets of metrics, with a new Ligand analysis track added to the existing Fit-to-Map, Coordinates-only, Comparison-to-Reference, and Comparison-among-Models tracks.

Global Fit-to-Map metrics included Map-Model Fourier shell correlation (FSC)^17^, Atom Inclusion^18^, EMRinger^7^, density-based correlation scores from TEMPy^19^, Phenix^20^ and Q-score^10^.

Overall Coordinates-only quality was evaluated using Clashscore, Rotamer outliers, Ramachandran outliers, and CaBLAM from MolProbity^9,21^, as well as standard geometry measures (e.g., bond, chirality, planarity) from Phenix^22^. Davis-QA, a measure used in critical assessment of protein structure prediction (CASP) competitions, was used to assess similarity among submitted models^23^.

Assessment teams contributed a wide variety of ligand-specific assessments (Table 2, ids **AT01-AT07**) including ligand, ligand environment, solvent, and RNA-specific analyses. AT01 used Mogul^24^ to evaluate ligand covalent geometry as implemented in the wwPDB validation process^2,4^, with inclusion of a novel composite ligand geometry ranking score^25^. AT02 evaluated model ligands using Coot^26^ and AceDRG^27^. AT03 evaluated RNA conformation with DNATCO^28,29^ and solvent atom placement around protein residues using water distributions^30,31^. AT04 analyzed ligand all-atom contacts with Molprobity Probescore^9^, and ion and water placements using UnDowser^32^. AT05 scored ligand placements using density fields derived from pharmacophore consensus field analysis^33^, a method utilized in computer-aided drug design to identify and extract possible interactions between a ligand–receptor complex based on steric and electronic features^34^. AT06 examined ligand strain energies using both molecular mechanics and neural net potential energy strategies^35–37^, where strain energy is the calculated difference in energy between the modeled conformation and the lowest energy conformation in solution. AT07 prepared Q-score analyses^10^ for model-fit-to-map of whole models, protein, ligands, and water, as well as ligand plus protein and/or nucleic acid polymer atoms in the immediate vicinity of the ligand (LIVQ). Assessor scores are available online at model-compare.emdataresource.org; results are briefly outlined below.

### Outcomes

**Figure 3:**
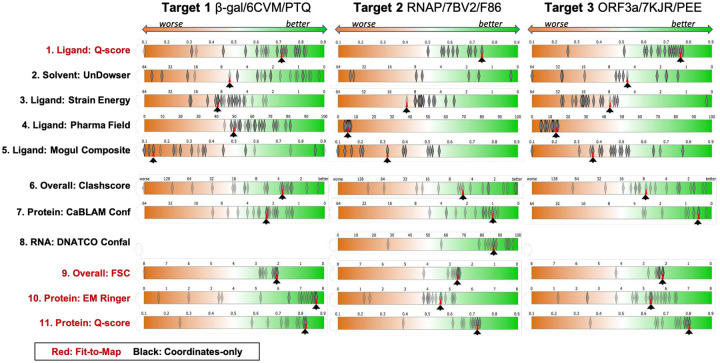
Model score distributions of selected assessments for Targets 1–3. Top 5 rows: ligand and solvent scores, bottom 6 rows: overall and protein-specific scores. Fit-to-Map based metrics have red labels; Coordinates-only metrics have black labels. Diamonds indicate individual scores of submitted models; red triangles (with supporting black arrows) indicate the scores of the reference models; in a few cases no score is available for the reference model. Each score distribution is plotted against an orange(left)-white-green(right) color gradient with orange indicating poorer scores, and green indicating better scores^8^.

The modeled ligands from each of the submissions are shown superimposed with their corresponding map density in Figure 2 **panels D-F**; selected ligand and whole-model score distributions are shown for all three targets in Figure 3. The full set of pipeline and assessment team scores and their definitions are provided in **Supplementary Data S3**. The superimposed views and score distributions demonstrate that the methods utilized by the modeling teams produced a range of ligand positions and conformations.

#### Overall model scoring.

With regards to overall Fit-to-Map evaluation, the majority of submitted models scored very similarly to PDB reference models for all targets, both in terms of the overall map-model FSC^17^ and protein Q-score^10^ (Figure 3, **rows 9 and 11**). For Targets 2 and 3, several teams modestly improved upon EMRinger score^7^ (Figure 3, **columns 2 and 3, row 10**). With regards to overall Coordinates-only evaluation, many teams were able to improve upon PDB reference models for all targets in terms of Clashscore^32^ and CaBLAM^32^, metrics that identify steric clashes and evaluate protein backbone geometry, respectively (Figure 3, **rows 6, 7**).

#### Ligand and ligand environment scoring.

Ligand and ligand environment evaluation methods were challenged by missing atoms in some submissions, the covalently bound ligand (Target 2), and presence of charged ligands (Targets 2 and 3). In terms of ligand-specific Fit-to-Map (Ligand Q-score), many teams made improvements relative to the PDB reference model of Target 1, but scored similarly or worse than the PDB reference of Targets 2 and 3 (Figure 3, **row 1**). In terms of covalent geometry (Mogul)^24,25^, many ligands in the submitted models were improved relative to references for Targets 1 and 3, while results were mixed for Target 2 (Figure 3, **row 5**). With respect to calculated ligand strain energy and pharmacophore ligand environment modeling, many of the submitted models were improved relative to references for Targets 1 and 2, but some poses were less favorable (Figure 3, **rows 3–4**). Ligand strain energy qualitatively should be less than 3 kcal/mol with minor relaxation using the sampling and scoring as described in Online Methods. Only a subset of submitting groups carefully considered treatment of ions (Extended Data Figure 5).

#### Nucleic Acid scoring.

Target 2’s RNA (a typical A-form double helix, with two unpaired nucleotides at the 5՛ end of the template strand) had close to expected geometries for most submitted models as assessed by DNATCO nucleic acid Confal scores^29^ (Figure 3, **row 8**). Values of torsion angles in the dinucleotide units assigned to DNATCO NtC classes agreed with expected distributions including sugar ring torsions that define pucker. Note that prior to running this Challenge, Target 2’s reference model (PDB 7bv2) had been re-versioned by the deposition authors and re-released by the PDB with several corrections to sequence, RNA conformation, and CaBLAM outliers^38^, thus limiting scope for model improvement.

#### Submitted Model rankings.

**Figure 4.**
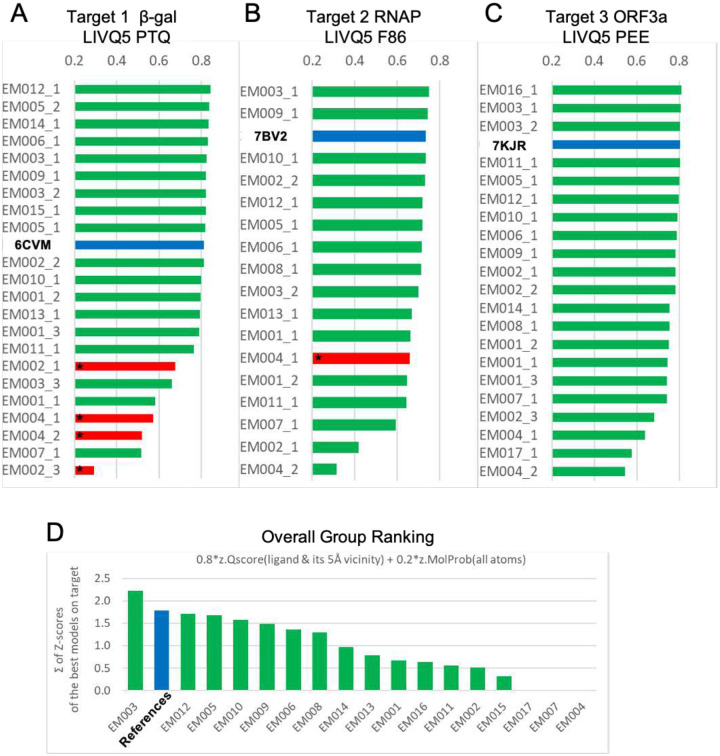
Model and modeling group rankings. (**A-C**) **LIVQ5** (**Ligand + Immediate Vicinity Q-score** ≤5Å) is plotted according to rank for each submitted model (labeled as participant group id, see Table 1, followed by model number) and for each reference model (labeled as PDB id). Models with good overall MolProbity (MP) scores (<3.0) are shaded green; those with poor MP scores (>3.0) are shaded red and starred; reference models are shaded blue and labeled in bold. Immediate vicinity includes all non-hydrogen provided in Extended Data Figure 2. (**D**) Ranking of Challenge participant groups based on the Fit-to-Map accuracy of ligands as shown in (**A-C**), and stereochemical plausibility, as described in the main text. Overall rank is calculated as the all-target sum of weighted z-scores for the best per-target models from the group (see equation in main text).

To evaluate and rank quality of ligand Fit-to-Map *within the context of the macromolecular complex*, we developed a novel score, the **Ligand + Immediate Vicinity Q-score (LIVQ)**, which averages Q-scores of non-hydrogen atoms of the ligand together with all non-hydrogen polymer atoms in the immediate vicinity of ligand. A distance cutoff of 5 Å was chosen to define the immediate vicinity of the ligand for model ranking purposes (**LIVQ5**, Figure 4A–C); extension to 10 Å yielded similar results (**LIVQ10**, Extended Data Figure 2). The results of the analysis show that for each target there are several models that exhibit very good model-to-map fit comparable to that of reference PDB-deposited models (Figure 4A–C, blue bars). Nine, two and three submitted models respectively on Targets 1–3 score better than the corresponding deposited reference model.

#### Group rankings.

Overall ranking of participating groups (Figure 4D) employed a combination of LIVQ5 and MolProbity score, itself a weighted function of clashes, Ramachandran favored, and rotamer outliers^9^. LIVQ5 was weighted higher than stereochemical plausibility, similar to the approach customarily used in CASP^39^:

$$\text{rank}=\sum_{\text{target}=1\ldots 3}\left(0.8*z.LIVQ5_{\text{target}}+0.2*z.\text{MolProbit}y_{\text{target}}\right)$$

where *z.metric* is the number of standard deviations relative to the mean of the score distribution for all models from each group on the selected target according to the selected metric. Overall, group EM003 (DiMaio) had the best relative performance by this ranking criterion, being the only group that outscored all deposited reference PDB models (Figure 4A–C).

#### Alternate group rankings.

The model-compare website Group Ranking calculator enables users to explore other possible ranking formulas: z-scores of up to 40 different individual metrics can be selected for inclusion with adjustable weighting. Extended Data Figure 3 illustrates an alternate ranking method based upon thirteen different metrics including ligand, ligand+environment, full model coordinates-only and full model fit-to-map. By this alternate method, five groups ranked higher than PDB reference models: EM010 (Chojnowski), EM008 (Emsley), EM012 (Palmer), EM003 (DiMaio), and EM009 (Moriarty), and one performed very close to reference, EM011 (Igaev).

#### Ligand Quality.

**Figure 5.**
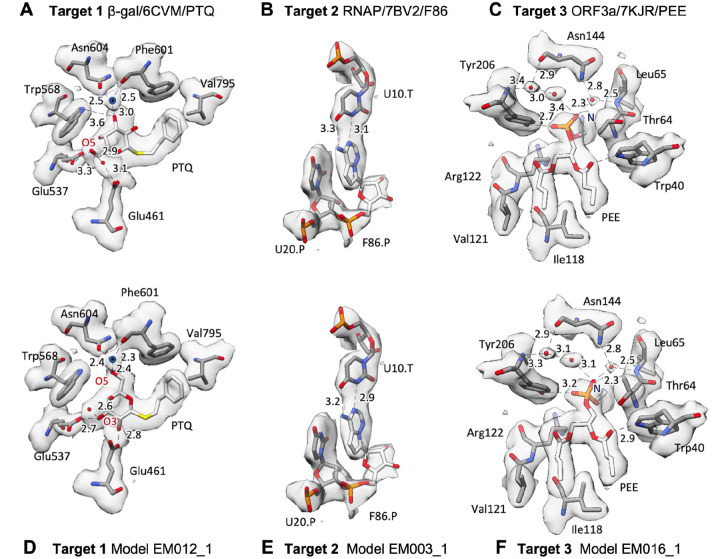
Visualization of ligands and surrounding atoms in deposited reference models and best-scoring submitted models (A,B,C) deposited reference models for Targets 1–3 as described in the main text. (D,E,F) best-scoring submitted models for each target. Modeled solvent atoms are shown as red spheres; a modeled ion in panels A,D is shown as a dark blue sphere. Numerical labels with dashed lines indicate atom-to-atom distances in Ångstroms.

The ligand environment for the reference models and the best submitted models is compared for each target in Figure 5.

For Target 1 (β-Gal, Fig 5 A,D), the PTQ ligand O5 atom connected to the sugar ring is situated at the bottom of the binding pocket in the reference model and in eight submitted models, whereas in the top-scoring model, as well as five other submitted models, the sugar ring is flipped with oxygen O5 situated at the top. The flipped ligand fits the density better and has more optimal interatomic distances to water and protein atoms for hydrogen-bonding, with O5 H-bonded to a coordinated water of the nearby magnesium ion (see Supplemental section S5). The density shape does not preclude the possibility that both original and flipped conformations are present, each with partial occupancy, and probescores for the two states are nearly identical (Extended Data Fig 4A).

For Target 2 (RNAP; Fig 5 B,E), the F86 ligand is very similar for the deposited and top-scoring model, though distances to base-paired U10 are slightly different. F86 probescores varied greatly across models, with the reference at 10.1, model EM008_1 at 39.9, and the worst model at −106.9 (Extended Data Figure 4). Many models did not correctly create the RNA polymer – F86 (remdesivir) covalent bond. In addition, only five models indicated partial occupancy for F86, yet the map density for F86 and its paired base is almost exactly half that of adjacent base pairs (Extended Data Fig 4B), indicating 50% occupancy.

In the case of Target 3 (ORF3 ion channel; Fig 5 C,F), the PEE ligand has similar interactions to nearby atoms and placed water molecules, though with slightly different interatomic distances. The head-group amino N atom (which has no close contacts within 4Å) points up in the deposited model but away from the camera view in the top-scoring model (Fig 5F). The long lipid tails of PEE have lower density, with confusingly interlaced and gapped connectivity that indicates disorder; the ensemble of all PEE ligand models shown in Fig 2F may be a more meaningful representation than any one individual model.

## Discussion

The selected targets for the Ligand Challenge are some of the first structures deposited and released into PDB that contain ligands modeled into cryo-EM maps with resolution of 3 Å or better. Our Challenge results revealed that a deposited PDB model’s ligand and local ligand environment may not be fully optimal in terms of concurrent Fit-to-Map and Coordinates-only measures. For all three targets and especially for Target 1, adjustments in the ligand and/or ligand environment could be made to the deposited reference model that improved one or more validation criteria, as demonstrated by several modeler groups. Most of the submitted models were in the “better” range, where tiny differences in measured scores become inconsequential. In our previous Challenge, we showed that overall Fit-to-Map and Coordinates-only metrics are orthogonal measures^8^; here we see that at a local level, ligand/ligand-environment Fit-to-Map and Coordinates-only metrics are similarly independent (Figure 3, Extended Data Figure 3B, **Supplementary Data S3**). In other words, ligands that fit quite well into density may not be optimized with respect to ligand coordinates-only validation criteria, and vice versa.

Based on our analyses and experiences running the Challenge, we make the following recommendations.

### Recommendation 1, regarding validation of the macromolecular models:

For ligand-macromolecular complexes, the macromolecular model should be subject to standard geometric checks as done for X-ray crystallographic based models^1^. These include standard covalent geometry checks and MolProbity evaluation, including CaBLAM, clashscore^9,21,32^. Sugar pucker and DNATCO conformational analysis^28,29^should be checked for nucleic acid components. The macromolecular model-map fit should be evaluated by EM Ringer^7^, Q score^10^, and FSC^17^. Serious local outliers (which usually indicate an incorrect local conformation) should be emphasized, rather than overall average scores.

The individual MolProbity scores, CaBLAM and clashscore have more utility for validation of protein conformation than overall MolProbity score which incorporates Ramachandran and side-chain rotamer quality, since cryo-EM model refinement includes these as restraints.

### Recommendation 2, regarding validation of ligand models:

Ligands in macromolecular complexes should conform to standard covalent geometry measures (bond lengths, angles, planarity, chirality) as recommended by the wwPDB validation report^2,4^. Additional checks that should be applied to ligands include fit to density using methods applicable to cryo-EM such as Q-score, occupancy (density strength, both absolute and relative to surroundings), and identification of missing atoms, including any surrounding ions.

Ligand energetics should also be examined. Ligand models should be assessed for their strain energy (the calculated difference in energy between the modeled conformation and the lowest energy conformation in solution) to identify improbable model geometries and lower energy alternatives^35,36^. Other methods can be used but may have different thresholds due to variation in absolute energy values. Strain energy calculations using neural net potentials offer speed close to force fields with the accuracy of QM calculations and are predicted to play a primary role in identifying accurate strain energies in the future. More research is needed to evaluate the overall utility of these deep learning novel methods.

### Recommendation 3, regarding validation of ligand environment:

The detailed interaction of the ligand with its binding site is of great importance and should be assessed by several independent metrics. Pharmacophore modeling^33^ is an optimized and time-tested energetic measure for how well the site would bind the specific ligand. LIVQ scores, introduced here, measure the density fit of the surrounding residues as well as the ligand itself. Probescore^32^ both quantifies and identifies specific all-atom contacts of H-bond, clash, and van der Waals interactions. All three types of measures should be taken into account. If the ligand model shows only weak interaction with its environment, the model is not right.

During the virtual wrap-up workshop, modelers and assessors shared their experiences and strategies to identify/assess the correct pose for the ligand based on the cryo-EM density maps. It was noted that the local map resolution for a ligand can be worse than the overall map resolution. As one objective measure, Q-scores were found to be lower for ligands in the best submitted models than for the nearby environment (Table 3). Factors that may affect resolvability of local ligand map features include incomplete occupancy, multiple conformations/poses present, regions of ligand flexibility or disorder, chemical modifications, and radiation damage.

### Recommendation 4, regarding organization of future Challenges:

Future cryo-EM Model Challenges should be organized similarly to the well-established CASP and CAPRI challenge events of the X-ray crystallography and prediction communities^23^, with incorporation of automated checks and immediate author feedback on all model submissions.

### Recommendation 5, regarding topics for future Challenges:

For future Challenge topics, consider validation of RNA models, including identification of RNA-associated ions, owing to the rapidly rising numbers of RNA-containing cryo-EM structures^40–42^. We also recommend maps determined in the 3.5-to-10 Å resolution range be considered as future targets to reflect the rapid rise in depositions of maps from subtomogram averaging of components in cell tomograms^43–45^. There are very few validation tools for that resolution range.

## Online Methods

### Challenge process and organization

The Ligand Model Challenge process closely followed the streamlined procedure adopted in the previous Model Metrics Challenge^8^. In the fall of 2020, a panel of advisors with expertise in cryo-EM methods, ligand modeling and/or ligand model assessment was recruited (J. Černý, P. Emsley, A. Joachimiak, J. Richardson, R. Read, A. Rohou, B. Schneider). The panel worked with EMDR team members to develop the challenge goals and guidelines, to identify suitable ligand-containing reference models from the PDB with cryo-EM map targets from EMDB, and to recommend metrics to be calculated for each submitted model.

The main stated goal was to identify metrics most suitable for evaluating and comparing fit of ligands in atomic coordinate models into cryo-EM maps with 3.0 Å or better reported overall resolution. The specific focus areas for assessor teams suggested by the expert panel were: (1) Geometry and fit to map of small molecules including ligands, water, metal ions, detergent, lipid, nanodiscs. (2) Model geometry (including backbone and side-chain conformations, clashes) in the neighborhood surrounding the small molecules. (3) Local model Fit-to-Map density per residue and per atom. (4) Resolvability at residue or atom-level. (5) Atomic Displacement parameters (B-factors) recommended optimization practice. A key question to be answered: How reliable are ligands/waters/ions built into cryo-EM maps? Can they be placed automatically or is manual intervention needed?

Modeling teams were tasked with creating and uploading their optimized model for each Target Map. The challenge rules and guidance were as follows: (1) Submitted models should be as complete and as accurate as possible (i.e., close to publication-ready), with atomic coordinates and atomic displacement parameters for all model components. (2) Submitted models must use the deposited PDB Reference Model’s residue, ligand, and chain numbering/labeling for all shared model components. (3) Ligands should ideally be deleted and refitted independently. (4) Additional polymer residues should be labeled according to the Reference Model’s sequence/residue numbering/chain ids. (5) If additional waters/ions/ligands are included, they should be labeled with unique chain ids. (6) If predicted hydrogen atom positions are part of the modeling process, hydrogens should be included in the submitted coordinates. (7) Models are expected to adhere to the reconstruction’s point symmetry (D2 for Target 1, C1 for Target 2, C2 for Target 3).

Members of cryo-EM and modeling communities were invited to participate in February 2021 and details were posted at challenges.emdataresource.org . Models were submitted by participant teams between March 1 and April 15. For each submitted model, metadata describing the full modeling workflow were collected via a Drupal webform (see **Supplementary Data S1, S2**), and coordinates were uploaded and converted to PDBx/mmCIF format using PDBextract^46^. Model coordinates were then processed for atom/residue ordering and nomenclature consistency using PDB annotation software (Feng Z., https://sw-tools.rcsb.org/apps/MAXIT) and additionally checked for sequence consistency, ligand atom naming, and correct position relative to the designated target map. Models were then evaluated as described below (see Model evaluation system).

In mid-April 2021, models, workflows and initial calculated scores were made publicly available for evaluation, blinded to modeler team identity and software used. In the period mid-April to mid-May, evaluators discovered several problems with the submitted models that blocked assessment software from completing calculations. The primary issue identified was inconsistent ligand atom naming. Approximately half of all submitted models had to be revised to make atom names consistent with the deposited reference models (see Challenge rule (2) above). Corrected coordinate files were provided by the submitting modeler teams, which were then re-processed as described above and re-released to evaluators.

A virtual 3-day (~4 hours/day) workshop was held in mid-July 2021 to review the Challenge results. All modeling participants were invited to attend remotely and present overviews of their modeling processes and/or assessment strategies. Recommendations were made for additional evaluations of the submitted models as well as for future challenges. Modeler teams, workflows and software were unblinded during the workshop.

### Data sources and Modeling

Target maps were obtained from EM Data Bank^47^. **Target 1** E. coli β-Galactosidase/PETG^12^: EMD-7770, **Target 2** SARS-CoV-2 RNA-dependent RNA polymerase/Remdesivir^13^: EMD-30210, **Target 3** SARS-CoV-2 ORF3a putative ion channel/phospholipid in nanodisc^15^: EMD-22898.

Table 1 summarizes the approach and lists the software used by each modeling team. Further details for each model can be found in **Supplement S2**. Modeling teams categorized their polymer modeling type as either *ab initio* (followed by optimization), optimized, or not optimized. Non-*ab initio* approaches made use of polymer coordinates from the following PDB entries. **Target 1**: 6cvm, 1jz7, 6tte. **Target 2**: 7bv2, 7b3d, 6×71, 3ovb. **Target 3**: 7kjr.

Submitted models were further categorized by ligand modeling type, either independently refit or optimized. Initial ligand coordinates and restraints were obtained from the PDB Chemical Component Dictionary (CCD)^48^, Crystallography Open Database (COD)^49^, or from a PDB entry. Ligand restraint generation software included BUSTER Grade (Global Phasing Ltd., Cambridge, UK), Phenix eLBOW^50^, CCP4 AceDRG^51^, PyRosetta^52^, AMBER Antechamber^53^, OpenBabel^54^, CHARMM CGenFF^55^, LigPrep (Schrödinger LLC, New York, USA), and CCP4 monomer library^56^. Restraints were not applied by teams using MD-based approaches.

*Ab initio* modeling software included ARP/wARP^57^, Mainmast^58^, Mainmastseg^59^, Pathwalker^60^, Rosetta^61^, Modeller^62^, and DeepTracer^63,64^. Model optimization software included CDMD^65^, Phenix^22^, REFMAC^66^, Servalcat^67^, ProSMART^68^, MDFF^69^, CryoFold^70^, Amber^53^, MELD^71,72^, Schrödinger (Schrödinger LLC, New York, USA). The program doubleHelix^73^ was used to assign RNA sequence and refinement restraints. Atomic displacement parameters (B-factors) were optimized for 32 of 61 models, with 23 applying individual atomic B-factors.

Participants made use of VMD^74^, Chimera^75^, ChimeraX^76^, Coot^26^, ISOLDE^77^, EMDA^78^ and PyMOL for visual evaluation and/or manual model improvement of map-model fit. Manipulation of map densities was carried out using CCP-EM^79^, EMDA, and LAFTER^80^.

### Model evaluation system

The evaluation pipeline for the 2021 challenge (model-compare.emdataresource.org) was built upon the basis of the 2019 Model Challenge pipeline^8,16^. Submitted models were evaluated for >70 individual metrics in four established tracks: Fit-to-Map, Coordinates-only, Comparison-to-Reference and Comparison-among-Models, plus a new Ligand track, created for comparison of ligand-specific scores (See **Supplementary Data S3**). Ligand and Nucleic-acid specific scores provided by Assessor teams (Table 2) were integrated into data tables alongside scores from the evaluation pipeline to enable comparisons and composite score generation.

### Pharmacophore Modeling

The Molecular Operating Environment platform (MOE) was used to score the placement of ligands. Starting from the model coordinates submitted by each group, the MOE QuickPrep application was used to prepare all-atom structures with hydrogens and atomic partial charges. For each target, an ensemble of structures consisting of all submitted models was input into the db_AutoPH4 application to produce pharmacophore consensus fields based on the ensemble. The pharmacophore consensus fields were then used to score the ligand poses of each submission. Additional details are provided in **Supplementary Data S4**.

### Strain energy calculations

Preparation: ligands were extracted from model files. For the T2 F86 ligand, strain energy was measured after deleting the covalent bond to the RNA polymer (SMILES:Nc(ncn1)c2n1c([C@]3(C#N)O[C@@H]([C@H]([C@H]3O)O)COP([O-])([O-])=O)cc2). For the T3 PEE ligand, all models were truncated to just the head group (SMILES:CCC(OC[C@@H](OC(CC)=O)CO[P@]([O-])(OCC[NH3+])=O)=O). Hydrogens were added using MOE/Protonate3D from the Chemical Computing Group.

Molecular Mechanics (MM) Forcefield Strain Energy: *predicted ligand energy* was calculated by minimizing each ligand structure using OpenEye/SZYBKI (MMFF94S with Sheffield solvation model) with a maximum RMSD of 0.6 Angstroms. Predicted *global minimum energy* was identified by sampling conformations using OpenEye/Omega and then minimizing each conformer structure using OpenEye/SZYBKI (MMFF94S with Sheffield solvation model) with no restraints, then selecting the conformer with the lowest minimized energy.

Neural Net Potential (NNP) Energy: *predicted ligand energy* was calculated by minimizing each ligand structure in an in-house implementation of the ANI neural net potential^37^ with a maximum RMSD of 0.6 Ångstroms. Predicted *global minimum energy* was identified by sampling conformations using OpenEye/Omega and then minimizing each conformer structure using the same in-house implementation of the ANI neural net potential with no restraints.

Reported scores are predicted strain energy as (*predicted ligand energy* - *global minimum energy*) in kcal/mol. NNP was only calculated for the T1 ligand as the method currently does not support atomic charges.

### Molecular Graphics

**Extended Data Figure 1.**
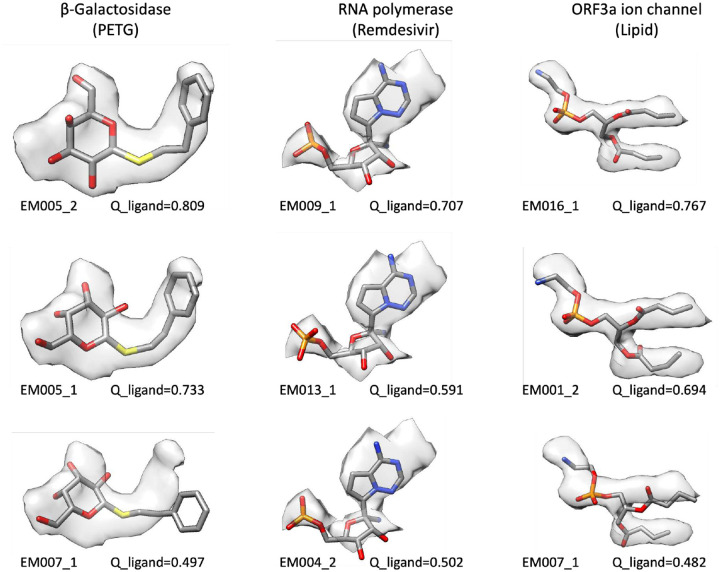
Selected submitted ligand models for each of the Challenge targets, labeled by team ID and model # (see Table 1), in order of decreasing ligand Q-scores (see Figure 3, row 1) from top to bottom. The portion of the map corresponding to the ligand is shown as a semi-transparent surface, along with the model of the ligand. Ligand Q-score is the average Q-score of all non-H atoms in the ligand. For each atom, the Q-score is measured by correlation of map density to the expected gaussian function, at points within 2 Å of the atom and closer to the atom than any other non-H atom in the model^10^. Higher-scoring ligand models fit better in the cryo-EM density than lower-scoring models.

Molecular graphics images were generated using UCSF Chimera (Figures 2, 5, Extended Data Figure 1).

### Classification of unique ligands in PDB introduced by Cryo-EM

Search of the Protein Data Bank via RCSB PDB’s data API^81^ identified 981 unique non polymer ligands/PDB Chemical Component Dictionary (CCD) ids in EM-derived PDB structures released through December 2021. Next, for each ligand, the PDB entry that first introduced the ligand/CCD id was identified. The 403 unique non-polymer ligands that were found to be introduced in structures determined by cryo-EM were then manually classified as enzyme modulators (substrates, inhibitors, agonists, co-factors), medically relevant drugs, lipids, photochemicals (e.g. carotenoids), peptides (amino-acid-based), reagents (buffers or labels), nucleotides, or steroids (fused rings).

## Extended Data

**Extended Data Figure 2.**
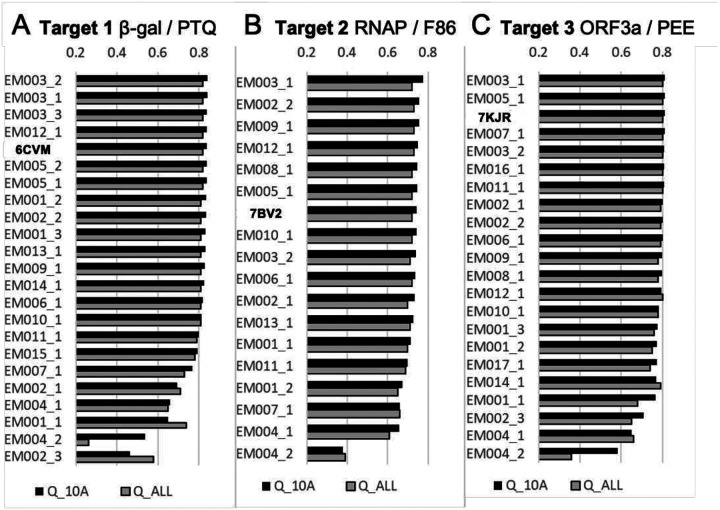
Q-score rankings for ligand + extended vicinity and for full models. (**A-C**) LIVQ10 (Ligand + extended vicinity ≤10 Å) Q-scores (black bars) and full model Q-scores (gray bars) are plotted for each submitted model and each reference model, with order according to ligand + extended vicinity rank. Reference model positions are highlighted with red arrows. Target/reference labels are as defined in the Figure 4 legend.

**Extended Data Figure 3.**
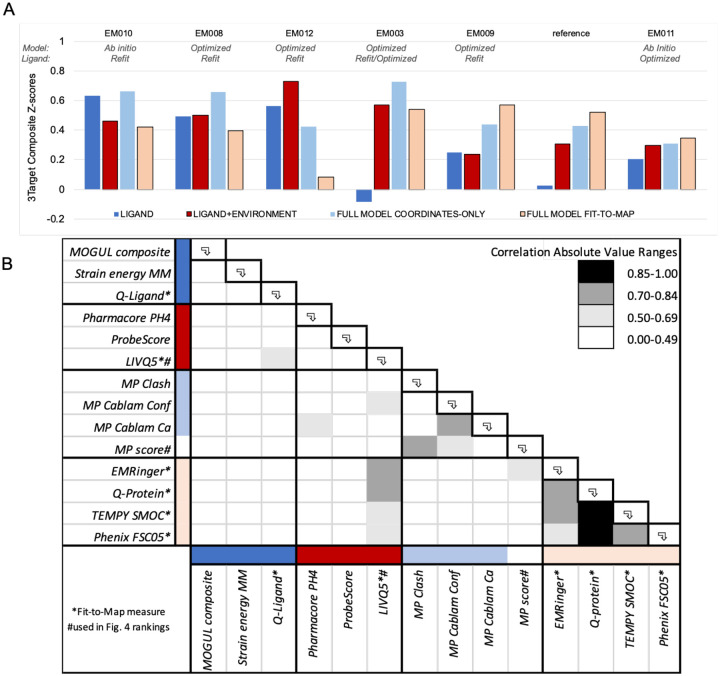
Alternative Group Ranking by sum of Ligand, Ligand+Environment, Full Model Coordinates-only, Full Model Fit-to-Map composite scores. (A) Group ranking (left-to-right) according to the sum of four composite z-scores, as described below. Only groups that submitted models for all 3 targets and have rank similar to or better than PDB reference models are shown. (B) Correlation table (n=64) of scores used to create z-scores and rankings in panel (A) and/or Figure 4. Group composite scores were calculated per team as follows. For each submitted model, and for each score type, a composite z-score was calculated. For each target (T1, T2, T3), the model submitted by that group with maximum composite z-score was selected for inclusion in the final average score over all targets. Ligand: z=(0.33*z.MogulComposite + 0.33*z.StrainEnergyMM + 0.33*z.Q-ligand) Ligand+environment: z=(0.33*z.Pharmacore + 0.33*z.Probescore + 0.33*z.LIVQ5) Full model coordinates-only: z=(0.25*z.Clash + 0.5*z.CablamConf + 0.25*z.CablamCa) Full model fit-to-map: z=(0.25*z.EMRinger + 0.25*z.Q-Protein + 0.25*z.TEMPySMOC + 0.25*z.PhenixFCS05)

**Extended Data Figure 4.**
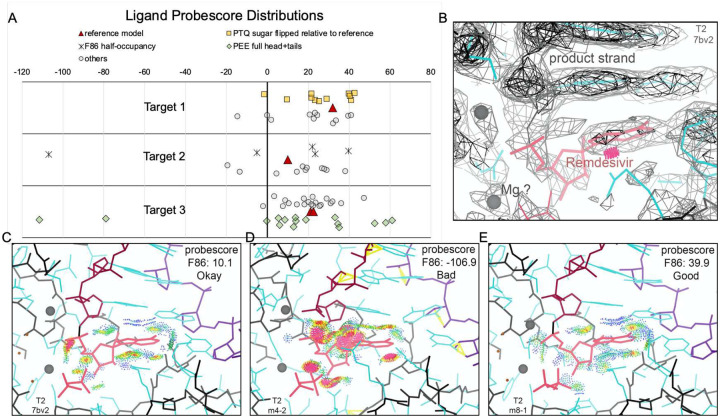
Ligand/Ligand Environment Probescores. (**A**) Molprobity Probescore^32^ distributions for ligands in Targets 1–3 (reference models: red triangles; submitted model scores are plotted as gray circles with following exceptions: Target 1, yellow boxes if PTQ sugar ring position was flipped relative to reference; Target 2, asterisk if F86 was set to half-occupancy; Target 3, blue diamonds if PEE was modeled as head-group+tails). Scores are plotted in horizontal axis lanes with small random vertical shifts to visually separate clustered points. Notably, score distributions have wide spreads independent of noted model features: PTQ sugar orientation, F86 occupancy, or PEE inclusion of tails–although for PEE the score distribution is noticeably broader when the larger and more variable tails are included. (**B**) T2 density map with reference model in the region of the F86 ligand^38^, showing half-strength density for the remdesivir ligand, implying that only half the molecules have covalently bound inhibitor. (**C-E**) T2 F86 + pyrophosphate ligand environments for the reference model (PDBid 7BV2), model EM004_2, and model EM008_1, respectively. All-atom contact dots are from Probescore, with all-atom clashes in hot pink and favorable H-bonds and vdW contacts in green and blue. Molecular graphics are shown in KiNG^83^.

**Extended Data Figure 5:**
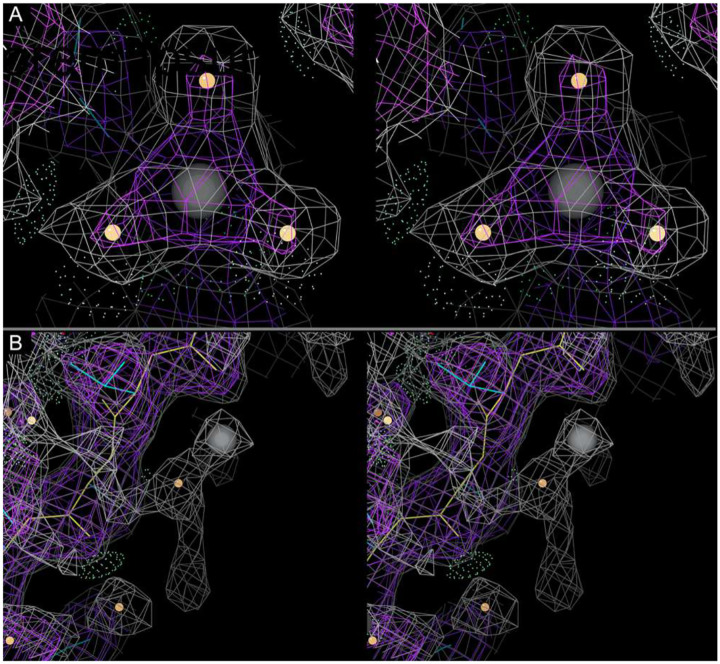
Evaluation of ions in submitted models (stereo images). (A) Target 1 6cvm reference model Mg A2002 (gray sphere) with water ligands (orange spheres), located near the PETG ligand, with density for classic octahedral coordination. Only six of 23 submitted Target 1 models included the Mg^2+^ and all three coordinating waters. Others had either only Mg^2+^, Mg^2+^ plus one or two waters, Mg^2+^ plus waters with zero occupancy, no atoms modeled, or atoms significantly displaced. (B) Some groups placed metal ions with weak justification, as exemplified by the Na^+^ (grey sphere) shown here in model EM005_1 for Target 3.

## Figures and Tables

**Figure 1.**
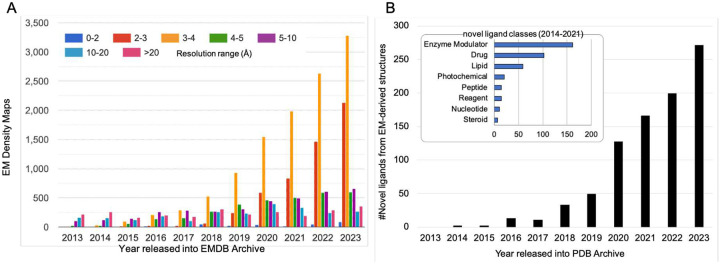
Growth of cryo-EM structures and novel ligands derived from them. (A) Cryo-EM maps released into the EM Data Bank (EMDB) archive by year and resolution range (source: www.emdataresource.org) up to the end of 2023. (B) Novel non-polymer ligands included in cryo-EM structures by year of release into the Protein Data Bank (PDB) through 2023. Inset: major categories of novel ligands found in cryo-EM-derived models (through 2021). See Online Methods for details.

**Table 1.** Modeling teams with number of models per target, approaches and software used.

ID	Modeling Team	T1	T2	T3	Polymer Modeling	Ligand Modeling	Ligand Restraints Software	Automati on level	Modeling Software
EM001	D. Kihara, G. Terashi, D. Sarkar, J. Verburgt	3	2	3	ab initio or optimized	refit or optimized	MD Force Field	partial	Mainmast, Mainmastseg, Rosetta PyMOL, Schrodinger, VMD, Chimera, MDFF
EM002	D. Si, S. Lin, M. Zhao, R. Cao, J. Hou	3	2	3	ab initio or none	refit	Phenix eLBOW	full	DeepTracer, Phenix
EM003	A. Muenks, F. DiMaio	3	2	2	optimized	refit	Phenix eLBOW, Open Babel	partial	Rosetta, Chimera
EM004	J. Cheng, N. Giri	2	2	2	ab initio	refit	PyRosetta	partial	Rosetta, Chimera, DeepTracer
EM005	G. Pintilie, M. Schmid, W. Chiu	2	1	1	none	refit	Phenix eLBOW	partial	Chimera
EM006	M. Baker, C. Hryc	1	1	1	ab initio	refit	Phenix eLBOW	partial	Pathwalker, Phenix
EM007	A. Perez, A. Mondal, R. Esmaeeli, L. Lang	1	1	1	optimized	optimized	PyRosetta, Antechamber, MD Force Field	partial	MELD, Amber, VMD
EM008	P. Emsley	1	1	1	optimized	refit	CCP4 AceDRG	partial	Coot, REFMAC
EM009	N.W. Moriarty, P. V. Afonine, C.J. Schlicksup, O.V. Sobolev	1	1	1	optimized	refit	Phenix eLBOW	partial	Coot, Chimera, ChimeraX, Phenix
EM010	G. Chojnowski	1	1	1	ab initio	refit	CCP4 mon lib	partial	ARP/wARP, ChimeraX, Coot, Isolde, Phenix, doubleHelix
EM011	M. Igaev, H. Grubmuller,. Pohjolainen, A. Vaiana	1	1	1	ab initio	optimized	MD Force Field	partial	Chimera, Modeller, VMD, CDMD
EM012	C. Palmer, R. Nicholls, R. Warshamanage, K. Yamashita, G. Murshudov, P. Bond, S. Hoh, M. Olek, K. Cowtan, A. Joseph, T. Burnley, M. Winn	1	1	1	optimized	refit or optimized	CCP4 AceDRG	partial	CCP-EM, Coot, EMDA, LAFTER, ProSMART, REFMAC, Servalcat
EM013	A. Singharoy, S. Mittal, A. Perez, D. Kihara, M. Shekhar, D. Sarkar, G. Terashi, C. Rowley, R. Esmaeeli, L. Lang, A. Mondal, A. Campbell	1	1		optimized	refit or optimized	CGENFF	partial	MDFF, CryoFold, MELD
EM014	W.-C. Kao, C. Hunte	1		1	optimized	refit	Grade (BUSTER), Phenix eLBOW	manual	ChimeraX, Coot, Isolde, Phenix
EM015	G. Schröder, L. Schäfer, K. Pothula	1			optimized	refit	MD Force Field	partial	CDMD
EM016	D. Kumar			1	optimized	refit	Phenix eLBOW	partial	Coot, Phenix
EM017	S. Weyand, S.C. Vedithi, T. Blundell, S. Brohawn			1	optimized	refit	Schrödinger Ligprep	full	Schrödinger
Totals		23	17	21					

**Table 2.** Ligand assessment teams and methods

Assessment Team ID	Team members	Assessment method
AT01	C. Shao	wwPDB validation report pipeline (Mogul)
AT02	P. Emsley	Coot Tools
AT03	B. Schneider, J. Černý	Nucleic acid conformations, protein hydration analysis
AT04	J.S. Richardson, C.J. Williams, V. Chen, D. Richardson	Contact analysis, probescore, occupancy, UnDowser, CaBLAM, visual examination
AT05	C.I. Williams, Chemical Computing Group Support Team	Pharmacophore density fields (PH4)
AT06	B. Sellers, A. Gobbi, S. Noreng, Y. Yang, A. Rohou	Molecular Mechanics Force Field Strain Energy (MM), Neural Net Potential Energy (NNP)
AT07	G. Pintilie, M. Schmid, W. Chiu	Q-score analysis

**Table 3.** Ligand and Ligand+environment Q-scores for submitted models with highest ligand Q-scores. Expected_Q is the expected Q-score for well-fitted models in maps at similar resolutions, based on analysis of a subset of publicly archived maps and models^82^. Q-scores well below the expected value indicate either that the map is not as well resolved as other maps at similar resolution, e.g. due to heterogeneity, or that the model is not optimally fitted to the map.

Target Map (Reported Resolution)	Model with highest ligand Q-score	Q_ligand (ligand atoms)	Q_near (atoms ≤5Å of ligand)	LIVQ5 (ligand +atoms ≤5Å of ligand)	Expected_Q at reported map resolution
T1 **β**-gal (1.9Å)	EM005_2	0.809	0.849	0.845	0.846
T2 RNAP (2.5Å)	EM009_1	0.707	0.735	0.731	0.690
T3 ORF3a (2.1Å)	EM016_1	0.767	0.819	0.812	0.791
